# Neuromechanical basis of region-specific differences and their implications for sport performance and injury prevention: a narrative review

**DOI:** 10.1007/s00421-025-05889-w

**Published:** 2025-07-25

**Authors:** Chrysostomos Sahinis, Ioannis G. Amiridis, Eleftherios Kellis

**Affiliations:** Laboratory of Neuromechanics, Department of Physical Education and Sport Sciences at Serres, Aristotle University of Thessaloniki, Agios Ioannis, 62110 Serres, Greece

**Keywords:** Compartmentalization, Regional differences, Muscle mechanics, Motor units, Neuromuscular control

## Abstract

This review aims to summarize current knowledge on regional variations in muscle structure and neural organization. It also examines how these differences affect muscle function and explores the current evidence for region-specific neuromuscular adaptations to training and injury/unloading in humans. Muscles exhibit substantial structural and functional heterogeneity along their length, reflected by regional variations in architecture, fiber type composition, sarcomere lengths, and tendon-aponeurosis structure. These differences potentially underpin distinct regional capacities for force production, contraction velocity, and energy storage. Additionally, compartmentalization within the neuromuscular system, characterized by spatially differentiated motor unit territories and region-specific activation, potentially allowing for specialized muscle function across varying tasks. However, current evidence predominantly relies on descriptive or animal studies, limiting direct translation into human-specific practices. Although preliminary findings suggest that region-specific adaptations occur following training, injury, or unloading, the practical implications for performance optimization or injury prevention remain speculative. Therefore, future longitudinal studies in humans are required to elucidate the functional relevance of these regional neuromuscular differences, to establish clearer mechanistic insights, and to determine whether such knowledge can guide training interventions and rehabilitation protocols to enhance performance and reduce injury risk.

## Introduction

Muscles are among the most flexible and adaptable tissues in the human body. This adaptability enables a wide range of movements necessary for daily activities, sports, and complex tasks (Bizzi et al. 1982; Kurtzer et al. 2005). The mechanical properties vary along the length of a muscle, reflecting a region-specific organization in which anatomically or functionally distinct segments exhibit different behavior (English et al. 1993). This regional differentiation supports the concept of muscle compartmentalization, where distinct regions within a single muscle or muscle group are specialized to perform different functions (English et al. 1993). This compartmentalization is not merely structural but also functional, influencing how muscles generate force and respond to various mechanical requirements (English et al. 1993; Higham and Biewener 2011).

The architecture of skeletal muscles dictate their function, governing their ability to produce force (Edgerton and Roy 1991; Lieber and Ward 2011; Narici et al. 2016). Muscles have been described in relatively simplistic terms, often assuming that their function is uniform along their length (Lieber and Friden 2000). Even within a single muscle there are significant architectural differences (English and Letbetter 1982; van Eijden and Raadsheer 1992; Blazevich et al. 2006; Kellis et al. 2010; Aeles et al. 2022). For instance, variations in fascicle length across muscle regions can result in localized differences in contraction speed and force production (Scott et al. 1996; Beck et al. 2022). Regional variations in tendon-aponeurosis interaction have also been found, influencing how force is distributed throughout the muscle (Finni et al. 2003; Kjær et al. 2009). Based on this evidence, it can be hypothesized that certain regions of a muscle could be responsible for force production, while others may function primarily in elastic energy storage.

Compartmentalization is also present in the nervous system, reflected in the spatial organization of motor pools within the spinal cord, where motoneurons project to specific regions of a muscle rather than innervating the entire muscle belly (Windhorst et al. 1989; English et al. 1993). Specific regions of a muscle could be preferentially activated depending on the required task, as some compartments may start to be activated sooner because of differences in neural drive and corticospinal input (English and Weeks 1984; Pratt and Loeb 1991; Farina et al. 2008; Watanabe et al. 2012b, a, 2013a; Gallina et al. 2013; Hegyi et al. 2018, 2019; Cohen et al. 2020). Additionally, proprioceptive feedback mechanisms are often localized within specific regions of a muscle, allowing for differential sensory control (Ting et al. 2015; Ting and Chiel 2017). Such neural partitioning not only governs precise force modulation but also contributes to the adaptability of the neuromuscular system under varying biomechanical conditions.

Another important factor affecting regional differences is whether a muscle is a monoarticular or biarticular. Biarticular muscles cross two joints contributing to energy transfer and coordination between joints, while monoarticular muscles generate torque at single joint (Lieber and Boakes 1988; van Ingen Schenau et al. 1994; Hof 2001). These functional differences impose distinct mechanical and neural demands, which may lead to region-specific variation in both muscle architecture and activation (Savelberg and Meijer 2003; Carroll and Biewener 2009). For biarticular muscles, neural control can be spatially coordinated to modify joint-specific contribution, leading to regional activation and fiber strain (Savelberg and Meijer 2003; Carroll and Biewener 2009).

**Fig. 1:**
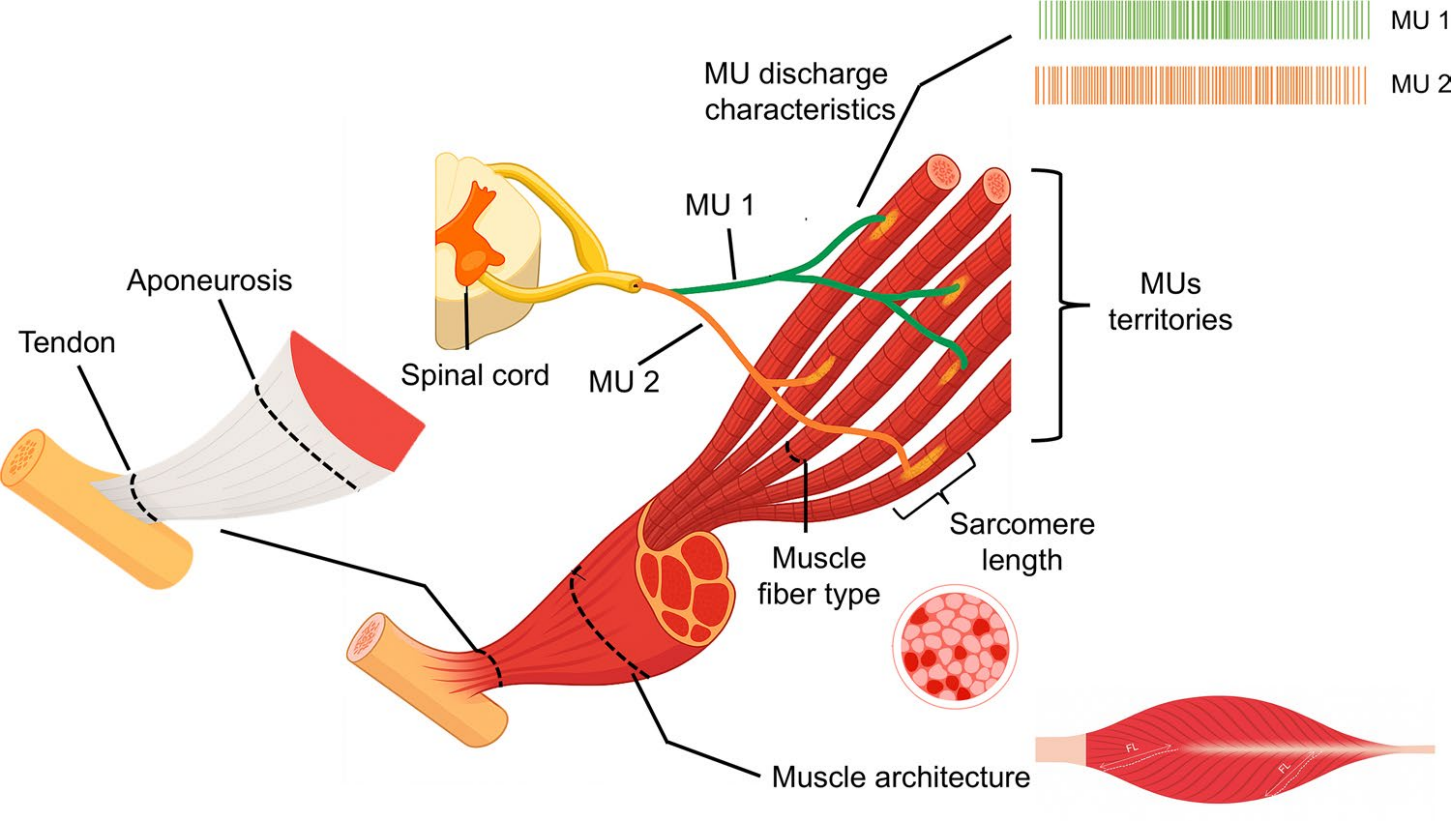
Illustration of the key neuromuscular factors that contribute to region-specific differences within a single muscle. Motor units (MU 1 and MU 2) innervate spatially distinct regions, reflecting compartmentalized neural control. Intramuscular differences in fiber type distribution, sarcomere length, and muscle architecture (e.g., fascicle length) may underlie regional heterogeneity in contractile function. Tendon-aponeurosis properties further influence local mechanical behavior

Despite the growing body of research on regional specialization in muscle-tendon unit  and the nervous system, many fundamental questions remain unanswered. For example, it is currently unknown to what extent the regional differences in structural and neural parameters exist and how these variations could affect injury risk and training outcomes. Most studies have focused on isolated aspects of either muscle architecture or neural compartmentalization without fully addressing how these two systems interact (Windhorst et al. 1989; English et al. 1993; Enoka 1995; Enoka and Fuglevand 2001; Monti et al. 2001; Liu et al. 2014). Additionally, there is a lack of consensus on the functional significance of regional differences with the previous studies have merely been descriptive (Windhorst et al. 1989; English et al. 1993; Enoka 1995; Enoka and Fuglevand 2001; Monti et al. 2001). This review aims to provide a synopsis of the current knowledge on the regional variation in structural and neural organization of the muscles (Fig. 1). We also discuss how these regional differences contribute to muscle function presenting the evidence on the region-specific neuromuscular adaptations following training interventions and injury/unloading in humans.

## Muscle specific factors

### Sarcomere length

The observation that length of sarcomeres varies along a muscle is not new (Hill 1953; Huxley and Peachey 1961). This non-uniformity in sarcomere length has been consistently observed in various species, including mice and humans, in both in vivo and in vitro settings (Edman and Reggiani 1984; Willems and Huijing 1994; Rassier et al. 2003; Llewellyn et al. 2008; Moo et al. 2016). This variability follows a consistent pattern across species, with distal fibers often exhibiting longer sarcomeres than proximal (Willems and Huijing 1994), suggesting regional specialization in force distribution and muscle function at rest (Edman and Reggiani 1984; Willems and Huijing 1994; Moo et al. 2016).

The available data on this topic in humans are currently limited (Walker and Schrodt 1974; van Eijden and Raadsheer 1992; Langenderfer et al. 2004; Ward et al. 2009; Lichtwark et al. 2018). In human studies, The sarcomere lengths were different  between proximal and distal parts of the muscle as well as compared to superficial and deep regions. The distal part of the muscles (including gastrocnemius, sartorius, extensor digitorum longus, soleus, masseter muscle and tibialis anterior) present greater sarcomere length than the proximal part (Walker and Schrodt 1974; Lichtwark et al. 2018) while deeper layers have shorter sarcomeres than superficial layers (van Eijden and Raadsheer 1992). Also, anatomical constraints (where the muscle narrows) near the origin and insertion may influence fascicle curvature and sarcomere orientation (Namburete and Wakeling 2012). These spatial factors could partly explain differences in sarcomere length across regions, particularly at the proximal and distal ends where geometric constraints are more pronounced. Recently, while Pincheira et al. (2022) and Andrews et al. (2024) reported sarcomere lengths in the central and distal regions of the biceps femoris (BF), both studies primarily analyzed adaptations over time rather than explicitly assessing region specific differences.

Are there any implications of the variability in length of the sarcomeres for understanding of muscle function? Due to the paucity of the available data, especially in humans, only assumptions can be made. Sarcomere length variability may influence the accuracy of models that describe muscle function. Traditional models of muscle function typically assume uniform sarcomere length throughout the muscle (Herzog et al. 2015) but latest studies have suggested that this assumption may be an oversimplification (Herzog et al. 2015; Moo et al. 2016, 2020). Typically, there is an optimal sarcomere length at which force production is maximized, and deviations from this optimal length (either by shortening or lengthening) may result in reduced force output (Gordon et al. 1966). Given the variability in sarcomere lengths between the proximal and distal regions within muscle, different regions may operate on different parts of the force–length curve (Ahn et al. 2003, 2018; Moo et al. 2020). This could lead to regional differences in force production during muscle contraction, especially during complex movements that involve changes in muscle length.

Moreover, the non-uniform distribution of sarcomere lengths within a muscle raises interesting questions about muscle adaptations. As muscles grow or adapt to training or injury, changes in sarcomere length might not occur uniformly, leading to shifts in how different regions contribute to overall muscle function (Lynn and Morgan 1994; Butterfield et al. 2005; Pincheira et al. 2022). For example, Pincheira et al. (2022) demonstrated that after eccentric training, the distal region of the BF exhibited significant increases in sarcomere length, while the central region did not, suggesting that muscle adaptation is region-specific and may influence the muscle’s overall functional capacity in a non-uniform manner. This finding may, in part, be explained by the mechanics of the Nordic hamstring exercise. Since the hip joint remains fixed during the movement, greater displacement likely occurs at the knee joint, particularly affecting the distal portion of the BF. Consequently, this region may experience greater mechanical strain, promoting localized sarcomere adaptation over the course of training.

Another important aspect is the impact of sarcomere length variability on muscle injury (Patel et al. 2004; Panchangam et al. 2008; Perrin et al. 2017). Muscle regions with longer sarcomeres may be more susceptible to strain injuries, as they are more likely to experience non-uniform strain and operate on the descending limb of the force–length relationship (Macpherson et al. 1997; Patel et al. 2004; Panchangam et al. 2008; Perrin et al. 2017). Understanding these differences could help in development of better prevention strategies for muscle strain injuries (Ralston et al. 2008). However, acquiring human sarcomere length data is technically and ethically challenging. Current in vivo sampling methods are highly localized and may not represent sarcomere variability across the entire muscle, further complicating generalization from available data (Lichtwark et al. 2018; Adkins et al. 2022).

### Fiber type composition

Muscles should be capable of both rapid movements and prolonged maintenance of posture, necessitating varying levels of power, and endurance to perform the daily tasks (Bizzi et al. 1982; Kurtzer et al. 2005). This functional diversity is achieved through a heterogeneous composition of muscle fibers, each characterized by distinct contractile and biochemical properties (Brooke and Kaiser 1970; Kernell 1998). Within individual muscles, there is considerable variation in fiber properties and fiber type composition often exhibiting regional specific organization (Elder et al. 1982; Garrett et al. 1984; Dahmane et al. 2005). These regional variations reflect a complex interaction of mechanical, functional, and developmental factors, often correlated with the muscle’s anatomical location and its specific functional demands (Eriksson and Thornell 1983; Korfage and Van Eijden 1999). Such regionalization has been consistently observed across species, including rats, rabbits, and humans, playing a critical role in the optimization of muscle performance (Armstrong and Phelps 1984; Kernell 1998).

Fiber composition can vary significantly between superficial and deep muscle regions, with fast-twitch fibers being more prevalent in superficial areas in both animal (James 1971; Grotmol et al. 2002) and human muscles (Elder et al. 1982; Dahmane et al. 2005). For instance, distal part of the vastus lateralis (VL), gastrocnemius, and biceps brachii often contain a higher proportion of fast-twitch fibers (Type II), whereas proximal part tend to have a greater proportion of slow-twitch fibers (Type I) (Johnson et al. 1973; Gollnick et al. 1974; Edgerton et al. 1975). The psoas major muscle also demonstrates a shift in fiber composition from cranial to caudal, with a decrease in Type I fibers and an increase in Type II fibers (Arbanas et al. 2009). This regionalization of fiber types within muscles potentially represents a strategic adaptation to meet specific functional demands, enhancing the muscle’s ability to perform both dynamic and static roles effectively.

Despite extensive research, the underlying mechanisms and functional implications of fiber type regionalization within muscles remain only partially understood, especially in humans (Kernell 1998). This limitation hinders our ability to draw definitive conclusions, as most available data are derived from animal models, they are interpreted primarily through various hypotheses rather than direct human evidence. Regions with a higher proportion of slow oxidative fibers are more active during endurance-based activities such as walking and postural control, while regions with a greater proportion of fast-twitch glycolytic fibers may be preferentially recruited during high-intensity, rapid force production tasks such as sprinting and jumping (Herring et al. 1979; De Ruiter et al. 1995; Monti et al. 2001). These potential variations could lead to exercise specific adaptations, where different types of exercises may preferentially target specific muscle regions. Additionally, these regional differences have been speculated to influence injury susceptibility with fast-twitch dominant regions potentially being more prone to strain injuries due to their lower fatigue resistance (Herring et al. 1979; De Ruiter et al. 1995; Monti et al. 2001).Overall, while fiber type regionalization appears to be a relevant factor in muscle function, the extent of its influence in humans remains largely theoretical. Future research should validate and extend these hypotheses to determine how regional differences in fiber type distribution contribute to movement mechanics and injury risk.

### Fiber architecture and strain

In classic biomechanical models, muscles are often treated as uniform structures with a singular, generalized function (Dong et al. 2002; Röhrle et al. 2012). These models, while useful for basic biomechanical simulations, fail to capture the complex reality of muscle architecture. They simplify the muscle to a degree that obscures the relation of fibers, pennation angles, and other architectural features that vary within the muscle (Zajac 1989; Delp and Loan 2000). Fiber architecture is different along the length of several compartmentalized such as ST (Woodley and Mercer 2005; Kellis et al. 2010; Kellis 2018) and soleus (Bolsterlee et al. 2018) or non-compartmentalized muscles such as VL (Blazevich et al. 2006), vastus medialis (VM) (Blazevich et al. 2006), BF (Woodley and Mercer 2005; Kellis et al. 2010; Kellis 2018) and MG (Aeles et al. 2022; Takahashi et al. 2022). For instance, in the MG, pennation angle is greater in the distal than the middle region, suggesting that the distal end of the muscle may be optimized for higher force production (Takahashi et al. 2022). This potentially reflects a broader pattern where different regions within the same muscle may be specialized for distinct functions.

These regional differences are not uniform across all muscles in different muscle groups. In some muscles, such as the VM, the fascicle angle increases as one moves distally, while in others, like the rectus femoris (RF), the angle decreases (Blazevich et al. 2006). Such diversity in architectural patterns across different synergistic muscles underscores the need for individualized analysis of muscle function rather than relying on generalized models (Blazevich et al. 2006). Region-specific differences have been also observed in hamstring muscles, with BF presenting longer fascicles and higher pennation angle proximally, while ST exhibiting the reverse pattern, with muscle thickness increasing from distal to proximal regions for both muscles (Kellis et al. 2010). While informative, these findings should be interpreted with caution, as two-dimension ultrasound cannot fully capture the three-dimension complexity of fascicle architecture, potentially limiting the accuracy of region-specific models (Aeles et al. 2022; Takahashi et al. 2022).

The functional significance of these regional architectural variations is substantial, as muscles are not uniform in their action but are instead specialized along their length to perform multiple roles. One hypothesis is that regions with greater pennation angle and larger PCSA are optimized for generating high forces, while other regions, where fibers are more aligned with the tendon’s line of action, may be efficient for force transfer (Spoor and van Leeuwen 1992; Lieber and Friden 2000). This suggests that muscles are designed to handle a range of mechanical demands, from generating force to precisely controlling movement (Lieber and Ward 2011). Additionally, variations in muscle architecture can cause significant differences in fiber strain within a muscle during contraction, resulting in region-specific hypertrophy in response to training. For example, muscle regions that experience higher strain may undergo greater hypertrophy, contributing to a non-uniform growth pattern within the muscle (Higham and Biewener 2011). This phenomenon has been observed in studies where proximal regions of muscles like the VM showed greater hypertrophy compared to distal regions under certain training interventions (Narici et al. 1996; Häkkinen et al. 2001).

The relationship between muscle architecture and fiber strain is complex (Azizi et al. 2008; Azizi and Roberts 2014). In pennate muscles, fibers are angled to the line of action, causing them to shorten and change angle during contraction, increasing force generation but reducing shortening velocity. These changes in pennation angle can amplify fiber strain, a phenomenon that has significant implications for muscle function (Azizi et al. 2008; Azizi and Roberts 2014). Such strain amplification is not limited to pennate muscles but can also occur in fusiform muscles, where differences in fiber curvature can lead to differential strain patterns between the inner and outer fibers (Daggfeldt 2006). These variations in strain are not just theoretical but have practical consequences for how different regions of a muscle contribute to overall muscle function (Higham and Biewener 2011). Regions experiencing greater strain may be more active during specific phases of movement, contributing more to force generation or work output (Azizi and Deslauriers 2014). Conversely, regions with lower strain may play a more supportive role, stabilizing the muscle or facilitating the transfer of force. Understanding these interactions is crucial for developing accurate models of muscle function that can predict how muscles behave under various conditions and potentially predicting their training or injury related adaptations (Azizi and Deslauriers 2014).

### Tendon morphology and mechanics

Tendons are stiff connective tissues that anchor muscles to bones, transmitting the forces generated by muscles to skeletal structures (Alexander 2002, 2011). While often perceived as simple, uniform structures, recent evidence suggests a much more complex reality (Ker et al. 1988; Matuszewski et al. 2012; Obst et al. 2014; Sahinis and Kellis 2024a). Tendons exhibit significant regional differences in both structural and mechanical properties (such as stiffness, stress and strain) (Farris et al. 2013; Obst et al. 2014; Reeves and Cooper 2017; Sahinis and Kellis 2024a). Providing insights into these regional variations is crucial, not only for understanding tendon mechanics but also for developing more effective treatments for tendon injuries.

At the most basic level, tendons are composed of collagen fibers arranged in hierarchical structures (Birk and Trelstad 1986; Kannus 2000; Franchi et al. 2007). These collagen fibers are grouped into primary, secondary, and tertiary bundles, which are surrounded by an extracellular matrix (Birk and Trelstad 1986; Kannus 2000; Franchi et al. 2007). This matrix is not homogenous but it varies in composition and turnover rate depending on its location within the tendon (Birch 2007). The outer matrix surrounding collagen bundles has been found to be more complex and to undergo more rapid turnover than the matrix within the bundles themselves (Thorpe et al. 2016a, b). This characteristic may contribute to the tendon’s ability to remain resilient and functional over time, despite the mechanical stresses it endures (Thorpe et al. 2016a, b). On a cellular level, tendons are equally diverse. The Achilles tendon of mice contains at least 13 different types of cells, including various fibroblasts responsible for maintaining the extracellular matrix (De Micheli et al. 2020). This cellular diversity suggests that different regions of the tendon might be specialized to handle different types of mechanical stress, a hypothesis supported by observations of differential gene expression across different parts of the tendon (Disser et al. 2022).

This regional specialization is further evidenced by the variation in tendon morphology such as the cross-sectional area (CSA). Significant changes in CSA along the length of Achilles tendon (Finni et al. 2003; Magnusson and Kjaer 2003; Farris et al. 2013; Obst et al. 2014) and hamstrings tendons have been observed (Sahinis et al. 2021; Kellis and Sahinis 2022; Sahinis and Kellis 2024a). Typically, the CSA of a tendon is greater in regions that experience higher mechanical loads, allowing these areas to withstand increased stress and reduce the risk of injury. For example, in human Achilles tendon, CSA is larger at the distal part compared to the more proximal and middle regions, which is thought to be an adaptation to the higher tensile forces encountered during activities like running and jumping (Magnusson and Kjaer 2003). This variation in CSA not only reflects the tendon’s load-bearing capacity but also influences its mechanical properties, such as stiffness and strength, across different regions (Alexander 2002). In line with these morphological differences, tendons mechanical properties (such as stiffness, stress and strain) are also not uniform but vary across different regions (Farris et al. 2013; Obst et al. 2014; Reeves and Cooper 2017; Sahinis and Kellis 2024a). Studies have shown that the young modulus, peak, and yield stress differ between the anterior and posterior portions of the human patellar tendon, indicating that these regions might respond differently to mechanical loading (Haraldsson et al. 2005; Hansen et al. 2009). Moreover, significant differences in transverse strain have been observed in both the Achilles (Farris et al. 2013; Obst et al. 2014; Reeves and Cooper 2017) and the hamstrings tendons (Sahinis and Kellis 2024a). Specifically, Sahinis and Kellis (2024a) found that the proximal regions of these tendons generally exhibited lower CSA strain compared to the middle and distal regions, suggesting that different parts of the tendon experience varying levels of deformation during loading. This non-uniform distribution of strain along the tendon length underscores the importance of considering these regional differences when evaluating tendon function. Also, recent studies have highlighted the contribution of subtendons to internal mechanical heterogeneity within the Achilles tendon (Finni and Vanwanseele 2023; Finni et al. 2025; Parkkola et al. 2025). The subtendons, which originate from individual triceps surae muscles, exhibit non-uniform displacement during loading aligning with the broader pattern of region-specific strain distribution observed across tendons (Finni and Vanwanseele 2023; Finni et al. 2025; Parkkola et al. 2025).

The regional heterogeneity of tendons has significant implications for both training and injury. Different parts of the Achilles tendon stretch to varying degrees during running or walking (Bojsen-Møller and Magnusson 2015; Franz et al. 2015; Slane and Thelen 2015; Adam et al. 2023), which may contribute to the localized nature of injuries like tendinopathy (Bojsen-Møller and Magnusson 2015). Therefore, it has been hypothesized that the uneven distribution of mechanical stress could explain the likelihood that tendon injuries often occur in specific regions rather than uniformly across the tendon (Bojsen-Møller and Magnusson 2015).

### Aponeurosis morphology and mechanics

In addition to regional differences in tendon morphology and mechanical properties, significant variations have also been observed in the aponeurosis. Aponeurosis is a stiff, sheet-like structure within the muscle–tendon unit which plays a crucial role in the transmission of force during movement (Eng et al. 2018; Bojsen-Møller and Magnusson 2019). Traditionally, aponeurosis was thought to function simply as an extension of the tendon, working in series with muscle fibers. This view may be overly simplistic and could lead to misunderstandings about its true function (Herzog 2017). Also, the aponeurosis contains a high density of muscle spindles and Golgi tendon organs (Maas et al. 2022), contributing to sensorimotor control during movement. This sensory input may support muscle gearing mechanisms, enabling dynamic adjustments in fascicle behavior during movement (Finni et al. 2023; Maas and Noort 2024). Regional differences in the morphological and mechanical properties of the aponeurosis have been identified along the length of the triceps surae (Finni et al. 2003; Shin et al. 2009; Oda et al. 2015; Shan et al. 2019) and hamstring muscles (Sahinis and Kellis 2024b, 2025), but similar data are lacking for other muscle groups. Specifically, aponeurosis of gastrocnemius and soleus is generally thicker in areas subjected to higher mechanical loads, such as near the muscle’s origin or insertion points (Oda et al. 2015). Similarly, aponeurosis stiffness decreases from the proximal to the distal regions of the MG muscle in human cadavers (Shan et al. 2019). This gradient is linked to the variation in thickness, with thicker regions being stiffer, providing essential resistance to elongation under tensile forces. The aponeurosis’s behavior during muscle contractions is further complicated by the type and intensity of muscle activity (Finni et al. 2003; Shin et al. 2009). Finni et al. (2003) highlighted that during submaximal isometric contractions, the middle region of the soleus aponeurosis lengthens while the distal region shortens. This indicates that the aponeurosis behaves in a region-specific manner, adapting to local mechanical demands rather than acting as a uniform passive structure. This nonuniform distribution is likely influenced by differences in muscle fiber orientation and connective tissue structure.

While direct measurements of regional differences in aponeurosis function remain limited, caution is warranted when interpreting its functional implications. Regional differences in the morphological and mechanical properties of the aponeurosis could significantly influence muscle function (Scott and Loeb 1995; Jaspers et al. 1999; Grega et al. 2020). These variations suggest that the aponeurosis is optimized for efficient force transmission, with thicker and stiffer regions near the muscle’s origin or insertion points resist deformation and effectively transmit force. Additionally, the nonuniform stiffness and strain distribution within the aponeurosis may contribute to its role in energy storage and release during movement, especially during activities like running and jumping (Scott and Loeb 1995; Jaspers et al. 1999; Grega et al. 2020). The regionalization of strain distribution observed during muscle contractions further support that different regions of the aponeurosis may have specialized functions depending on the type and intensity of muscle action.

## Neural specific factors

### Motor unit territories

In certain animal muscles, motor unit territories are compartmentalized within specific regions of the muscle, often separated by connective tissue (English and Weeks 1984; English et al. 1993). This compartmentalization potentially allows for more refined control over muscle force. In the cat lateral gastrocnemius (LG) muscle, motor unit territories are confined to distinct compartments, enabling selective activation of these compartments to control the direction of force generation (English and Weeks 1984). Similarly, in the highly compartmentalized rabbit masseter muscle, motor unit territories are restricted to small regions within the muscle, with territory sizes ranging from 1 to 10% of the muscle’s mid-belly cross-section (Vaughan and Goldspink 1979). In long strap-like muscles such as the cat sartorius, motor unit fibers are confined to specific, often asymmetrically distributed subvolumes along the muscle from origin to insertion, with individual motor units restricted to either the anterior or medial head and occupying 9% to 12% of the muscle volume (Vaughan and Goldspink 1979). This asymmetrical distribution of motor unit territories allows for differential activation of muscle regions, which may be important for complex movements that require the coordinated action of different muscle parts.

Current knowledge regarding region-specific differences in motor unit territories within human muscles remains limited (Enoka and Fuglevand 2001). Motor unit territories are often confined to limited regions within the muscle (Buchthal et al. 1959; Vieira et al. 2012, 2015; Gallina and Vieira 2015; Yu et al. 2016; Gallina et al. 2017). In the VM, motor unit territories are localized to specific regions along the muscle’s proximal-to-distal axis (Gallina and Vieira 2015). This regionalization is associated with differences in muscle fiber orientation, pennation angle, and fiber length, which influence the direction of force exerted by different regions of the muscle (Vieira et al. 2012, 2015; Gallina and Vieira 2015; Gallina et al. 2017).

The localization of motor unit territories within a muscle has significant functional implications (Monti et al. 2001) but direct evidence of human muscles and their relationship with human movement is scarce, limiting conclusions to hypothetical assumptions (Enoka and Fuglevand 2001). The regionalization of motor unit territories allows the CNS to distribute neural inputs to motoneurons based on the location of the muscle fibers they innervate. This arrangement may enable the CNS to selectively activate different regions of a muscle in response to specific sensory inputs, such as those from muscle spindles or stretch reflexes has been defined as reflex partitioning (Windhorst et al. 1989). For example, in the VM, the regionalization of stretch reflexes has been proposed as a mechanism by which the CNS can coordinate the activation of muscle regions with different mechanical properties (Gallina et al. 2017). This selective activation may be essential in situations where precise control of muscle force is required, such as during rapid directional changes in movement or in response to sudden perturbations. In highly compartmentalized muscles, such as the cat ST, the differential activation of motor unit territories within different compartments can result in complex mechanical interactions between compartments. For instance, when one compartment of the ST is activated, the resulting tension can cause lengthening in the adjacent, passive compartment, even though the overall muscle length remains unchanged (Nichols and Houk 1976). These interactions highlight the importance of motor unit territory localization in coordinating muscle function during movement. However, contrasting evidence suggests that compartmentalization may have limited functional significance. Bodine et al. (1982) found that despite the anatomical separation of the cat ST into proximal and distal compartments, fiber type composition, CSA, and contractile properties were similar, indicating that whole-muscle mechanics drive function rather than localized activation. Similarly, minimal regional variations in fascicle strain in fusiform muscles has been observed, suggesting that fiber architecture alone governs regional strain distribution without requiring independent compartmental control (Azizi and Deslauriers 2014). These findings challenge the assumption that compartmentalization significantly modulates strain and force transmission in parallel-fibered muscles like the ST.

### Motor unit discharge characteristics

Recent studies have highlighted the significance of regional differences in motor unit activity along the muscle length, particularly between the proximal and distal regions of a muscle (ter Haar Romeny et al. 1982, 1984; Keen and Fuglevand 2004; Reilly et al. 2004; Winges and Santello 2004; Harris et al. 2005; McIsaac and Fuglevand 2007; Sahinis et al. 2024). These differences, which have been observed in both animal and human studies, reveal a more complicated picture of neuromuscular control than previously understood (Fuglevand 2011; Heckman and Enoka 2012). These neuromuscular compartments exist in some, but not all, muscles, allowing independent activation of distinct regions (English and Weeks 1984; Keen and Fuglevand 2004; Harris et al. 2005; Sahinis et al. 2024). This challenges the assumption that muscle function can be inferred solely from skeletal attachments, as both architecture and innervation must be considered. For example, the biceps brachii has multiple neuromuscular compartments, with unique motor unit populations, one engaged during elbow flexion and another during combined flexion and supination (ter Haar Romeny et al. 1982; Van Zuylen et al. 1988). Also, independent neural drive and distinct motor unit discharge characteristics have been observed for ST muscle in humans, with the distal compartment influenced more the force variability during knee flexions (Sahinis et al. 2024). The coordination of specific muscles during movements has traditionally been attributed to a “common drive” that uniformly influences all motoneurons within a muscle (De Luca and Erim 1994; Iyer et al. 1994; Farina and Negro 2015; Negro et al. 2016) However, this view has been challenged by more recent findings (Hug et al. 2023). Studies have demonstrated that different regions of a muscle can have varying motor neuron branching patterns, which contribute to region-specific activation (Higham and Biewener 2011). Region specific activation has been observed in both animal (English and Weeks 1984; Pratt and Loeb 1991) and human studies (Farina et al. 2008; Watanabe et al. 2012b, 2012b, 2013a; Gallina et al. 2013; Hegyi et al. 2018, 2019; Cohen et al. 2020). For example, Watanabe et al. (2012b) demonstrated a task-dependent spatial distribution of electromyography (EMG) amplitude in the human RF muscle, revealing that hip flexion predominantly activated the proximal region, while knee extension primarily engaged the central and distal regions. Such differential activation suggests that even within a single muscle, different parts may be specialized for distinct functions. It is worth noting that during dynamic movements (Hegyi et al. 2018, 2019), shifts between the muscle and the surface electrodes can affect the accuracy of high density EMG recordings. Therefore, regional differences in activation, especially under non-isometric conditions, should be interpreted with some caution due to inherent limitations of surface EMG.

The partitioning of motor neuron pools within the spinal cord is one proposed mechanism for the observed regional differences in motor unit discharge characteristics (Windhorst et al. 1989; English et al. 1993). In animals, retrograde tracing studies have shown that motor neurons innervating different compartments of a muscle, such as the LG and MG, are organized into distinct nuclei within the spinal cord (Weeks and English 1985). These compartmental nuclei extend over a substantial part of the rostrocaudal axis but are typically grouped in specific regions corresponding to the compartments they innervate (Hoffer et al. 1987). This organization suggests that motor neurons innervating proximal compartments are primarily located in the rostral parts of the motor nucleus, while those innervating distal compartments are found more caudally. This topographical differentiation within the motor nucleus could explain the different activation of proximal and distal muscle compartments, potentially allowing the independent control of different muscle regions based on their functional demands. Another potential mechanism is the divergence of synaptic inputs to motor neurons innervating different muscle compartments. Some studies have indicated that last-order synaptic inputs may not be entirely separated, leading to partial overlap in the activation of motor neurons across different compartments (Botterman et al. 1983a, b; Vanden Noven et al. 1986; Hamm et al. 1987). This overlap could limit the ability to differentially activate specific muscle compartments, although it also provides a level of redundancy that could be beneficial in certain functional contexts, such as tuning muscle output to apply force in a coordinated manner.

The current evidence on regional variations in motor unit discharge characteristics is limited to isometric conditions, restricting the ability to draw definitive conclusions about their relevance to sport-specific movements. As a result, any extrapolations must rely on hypotheses derived from available animal data. The differential activation of muscle compartments can influence the overall force vector generated by the muscle, thereby affecting movement efficiency (Schieber 1993; Carrasco et al. 1999). This is essential in muscles that are involved in multi-joint movements, where precise control of force direction is crucial. Furthermore, the ability to selectively recruit different muscle compartments based on the task allows for greater flexibility and adaptability in motor performance. For example, in the human biceps brachii, the ability to differentially activate motor units during flexion versus supination enables the muscle to perform both functions effectively, even though they require different force vectors (ter Haar Romeny et al. 1982, 1984). Despite the advances in our understanding of regional differences in motor unit discharge characteristics, several questions remain unanswered. For example, the extent to which these differences are influenced by factors such as training and injury/unloading is currently unexplored.

## Region specific adaptations of the neuromuscular system

The following section focuses on the region-specific neuromuscular adaptations within the lower limb muscles in response to training and injury/unloading. Lower limb muscles are prioritized due to their critical role in athletic performance and sport-specific activities. These muscles not only exhibit substantial structural and functional heterogeneity, but they are also particularly susceptible to injury owing to their frequent exposure to high mechanical loads.

### Exercise intervention

#### Musculoskeletal adaptations

The current evidence indicates that resistance training induces region-specific adaptations in muscle size and architecture, with findings varying based on exercise modality, intensity, task type (single-joint vs. multi-joint) and targeted muscle groups (monoarticular vs biarticular muscles). A detailed summary of key studies examining these adaptations is provided in Table 1. The studies comparing concentric and eccentric knee extensions have shown greater increases in CSA at distal compared to proximal regions in the VL following eccentric training, whereas concentric training elicits more uniform hypertrophic responses (Seger et al. 1998; Franchi et al. 2014; Benford et al. 2021). Similarly, squat depth influences regional hypertrophy, with deep squats promoting significant CSA increases across all quadriceps regions, while shallow squats primarily enhance proximal CSA (Bloomquist et al. 2013). Additionally, Nordic hamstring exercises appear to preferentially increase fascicle length and sarcomere length in the distal BF, with limited changes in the mid-region (Pincheira et al. 2022; Andrews et al. 2024). In contrast, variations in knee flexion angle during leg extensions result in differential RF hypertrophy, with greater CSA increases observed in the distal region when training at 40° compared to 90° hip flexion (Larsen et al. 2024). Collectively, these findings underscore the complexity of region-specific muscle adaptations, reinforcing the need for further research to optimize training strategies for targeted exercise programs aiming to improve sport performance or to reduce injury risk.

**Table 1:** Summary of key studies that have examined region-specific muscle adaptations following exercise intervention. Table includes participant characteristics, training modalities, exercise parameters, and primary findings, with a focus on how different interventions influence hypertrophy across different regions of the lower limb muscles

Author	Participants	Training Modality	Reps × Sets	Intensity	Duration (weeks)	Frequency (days/week)	Results
Ahtiainen et al. (2003)	8 Trained M, 8 Untrained M	KE vs LP	3–15 × 3–6	50–80% 1RM	21	2	[Quadriceps] Untrained: CSA increased significantly at MID and DIS regions; Trained: No significant increase in any region
Andrews et al. (2024)	12 YM	NHE	4–5 × 6–8	Bodyweight	9	3	[BFlh] Significant change in FL and SL in the DIS compared to the MID region
Benford et al. (2021)	16 YM	CON vs. ECC KE	4 × 8	Maximal	5	2	[VL] CON: CSA and the PA increased significantly in the MD compared to the DIS region, FL showed a small increase at the MID region; ECC: the CSA increased significantly in the DIS compared to the MID region, FL and PA increased uniformly
Blazevich et al. (2003)	15 YM, 8 YF	Squat, Forward Hack Squat, Sprint/Jumping	4–6 × 6–10	Progressive (~ 70–85% 1RM)	5	3	[VL RF] MT increased significantly in the PROX compared to the DIS region; Region specific changes for PA and FL
Blazevich et al. (2007)	12 YM, 12 YF	CON vs ECC Isokinetic KE	4–6 × 6	Maximal	10	3	[VL RF] Uniform adaptations in CSA; [VM VI] CSA increased significantly at the DIS compared to the PROX region
Bloomquist et al. (2013)	17 YM	Deep Squat vs. Shallow Squat	3–5 × 3–10	Progressive	12	3	[Quadriceps] Deep Squat: CSA increased significantly at all measured regions; Shallow Squat: The CSA increased significantly only at the PROX region; [Hamstrings] Deep Squat: CSA increased significantly only in PROX site; Shallow Squat: No significant changes in CSA
Earp et al. (2015)	36 M	Strength vs Power training	3 × 3–8, 5–7 × 5–6	75–90% 1RM, 0–30% 1RM	8	3	Strength training: Greater hypertrophy in proximal VL, VM, and VI; Power training: Greater hypertrophy in distal VL and VI
Ema et al. (2013)	11 M	KE (CON/ECC)	8 × 5	80% 1RM	12	3	[VL, RF] The relative increase in CSA was significantly greater in DIS compared to PROX region; [VM] No change in CSA
Enes et al. (2024)	24 YF	Front Squat vs. Back Squat	2 × 6–12	Progressive loads by 20%	12	2	[VL] Uniform changes in CSA regardless of squat variation
Franchi et al. (2014)	12 YM	ECC vs CON Leg Press	4 × 8–10	80% 1RM	10	3	[VL] CSA increased significantly in the DIS region for the ECC compared to CON training while the opposite was observed for the MID region
Häkkinen et al. (2001)	10 OF	KE vs LP	5–20 × 3–6	40–80% 1RM	21	2	[VL] CSA increased significantly in the PROX to MID region; [VM] CSA increased significantly in the MID to DIS region; [VI] More uniform adaptations in CSA; [RF] CSA increased significantly in the PROX region
Häkkinen et al. (2002)	21 MAF	KE vs LP	5–20 × 3–6	40–80% 1RM	21	2	[VL and VM] Changes in CSA occurred predominantly in MID and DIS regions; [RF] Changes in CSA at PROX and MID regions; [VL] FL increased in the MID and DIS regions
Higbie et al. (1996)	35 YF	CON vs ECC Isokinetic KE	10 × 3	Maximal	10	3	[VL] CSA increased in MID and DIS regions; [RF] CSA increased in PROX
Hisaeda et al. (1996)	11 YF	High-volume (HV) vs Low-volume (LV) KE	HV: 4–5 × 15–20; LV: 8–9 × 4–6	HV: ~ 50–60% 1RM; LV: ~ 80–90% 1RM	8	3	[VL] HV: CSA changed significantly at PROX region. [RF] LV: CSA increased at DIS region; [VM] No significant changes in CSA
Housh et al. (1992)	13 YM	CON Isokinetic KE	10 × 6	Maximal	8	3	[VI, VL, BFlh] CSA increased MID region; [VM, SM] No change in CSA; [RF, ST] CSA increased in all regions
Housh et al. (1998)	9 YM	KE (CON)	3–4 × 8–12	80% 1RM	8	3	[Quadriceps] CSA increased significantly in MID region but less in the PROX and DIS regions
Hudelmaier et al. (2010)	16 MAF	Resistance training	Not reported	Progressive	12	3	The CSA increased at the PROX region for the quadriceps, hamstrings and adductors
Larsen et al. (2024)	22 YM	Leg Extension (40° vs. 90° Hip Flexion)	3–4 × 10–20	Progressive	10	2	[RF] MT increased significantly in the DIS compared to the PROX region; [VL] MT remained unchanged
Maeo et al. (2021)	13 YM, 7 YF	Seated vs. Prone Leg Curl	5 × 10	70% 1RM	12	2	[BFlh] CSA increased in PROX and DIS region for seated leg curl compared to prone leg curl; [ST] CSA increased only PROX region for seated leg curl compared to prone leg curl
Matta et al. (2015)	35 YM	Conventional vs Isokinetic KE	3 × 9–11, 3 × 10	Progressive	14	2	[RF] CSA and the MT increased for the DIS region for both training modalities; FA increased in the DIS compared to the PROX region only for the conventional training group
McMahon et al. (2014)	11 YM, 10 YF	Lengthened vs Shortened KE	3–4 × 8–10	Lengthened: 55% 1RM; Shortened: 80% 1RM	8	3	[VL] Significant changes at CSA only in DIS region
Melnyk et al. (2009)	11 YM, 10 YF and 11 OM, 11 OF	Unilateral KE	5 × 5–20	Progressive (5RM)	9	3	[Quadriceps] YM, OM and OW: CSA increased in PROX, MID, and DIS regions after strength training; YW: CSA increased only in MID and DIS regions
Narici et al. (1989)	4 YM	Isokinetic KE	10 × 6	Maximal	8	4	[Quadriceps] CSA increased in the PROX region compared to the DIS region
Narici et al. (1996)	7 YM	KE (CON/ECC)	8 × 6	80% 1RM	24	3	[Quadriceps] CSA increased in PROX and DIS region compared to the MID region
Noorkõiv et al. (2014)	16 YM	Isometric KE	5 × 5	Maximal	6	3	[Quadriceps] Region specific changes for CSA and FL
Pedrosa et al. (2022)	45 YF	KE with Partial and Full ROM	3–6 × 7	60% 1RM	12	3	[RF VL] Initial Partial ROM: CSA increased in MID regions; Full ROM: Uniform adaptations in CSA; Final Partial ROM: TCSA increased in PROX regions
Pincheira et al. (2022)	7 YM, 3 YF	NHE	4 × 6–8	Bodyweight	3	3	[BFlh] FL and SL increased DIS region, no change in MID region
Plotkin et al. (2023)	11 YM, 23 YF	Hip Thrust vs. Back Squat	3 × 8–12	Progressive	9	2	[Gluteus] Uniform changes in CSA for both exercises
Reeves et al. (2004)	4 OM, 5 OW	KE (CON/ECC) + LP (CON/ECC)	2 × 10	Progressive: 60–80% of 5RM	14	3	[VL] CSA increased significantly at MID region
Schott et al. (1995)	1 YM, 6 YF	Isometric intermittent vs continuous KE	IC: 10 × 4 CC: 4 × 1	70% MVC	14	3	[Quadriceps] No region-specific adaptations in CSA
Seger et al. (1998)	10 YM	CON vs ECC Isokinetic KE	10 × 4	Maximal	10	3	[Quadriceps] CON: CSA did not change in MID and DIS regions; ECC: CSA increased only in DIS region
Seynnes et al. (2007)	5 YM, 2 YF	KE (CON/ECC)	7 × 4	Maximal	5	3	[RF] CSA increased in PROX, MID, and DIS regions; [VL] CSA increased in PROX and DIS regions; [VM VI] CSA increased only in DIS region
Smith and Rutherford (1995)	5 YM, 5 YF	CON vs ECC Isokinetic KE	10 × 4	10RM	20	3	[Quadriceps] CSA increased in the PROX region for both CON and ECC
Valamatos et al. (2018)	19 YM	KE with Partial and Full ROM	5 × 6, 5 × 10	Equalized TUT	15	3	[VL] CSA increased only in MID and DIS regions
Zabaleta-Korta et al. (2021)	34 YM	Smith Machine Squat vs LE	4 × 12	To failure	5	3	[RF] LEG group: CSA increased at PROX, MID and DIS regions; [VL] SMTH group: CSA increased only MID region

YM, Young males; YF, Young females; MAF, Middle-aged females; OF, Older females; OM, Older males; MA, Middle-aged; KE, Knee extension; LP, Leg press; NHE, Nordic hamstring exercise; CON, Concentric training; ECC, Eccentric training; ROM, Range of motion; HV, High-volume training; LV, Low-volume training; IC, Intermittent isometric contraction; CC, Continuous isometric contraction; CSA, Cross-sectional area; MT, Muscle thickness; FL, Fascicle length; PA, Pennation angle; FA, Fiber angle; SL, Sarcomere Length; PROX, Proximal region; MID, Middle region; DIS, Distal region; ST, Semitendinosus; BFlh, Biceps femoris long head; SM, Semimembranosus; VL, Vastus Lateralis; VM, Vastus Medialis; RF, Rectus Femoris; VI, Vastus Intermedius; TUT, Time Under Tension

Moreover, resistance training leads to site-specific changes in the tendons, including nonhomogeneous adaptations of the morphological and mechanical properties over their length (Kongsgaard et al. 2007; Westh et al. 2008; Seynnes et al. 2009; Lambrianides et al. 2024). Table 2 summarizes key studies investigating region-specific tendon adaptations in response to different training modalities. Following resistance training the patellar tendon, has resulted in significant proximal and distal hypertrophy with increases in CSA of ~ 6% and ~ 4%, respectively, while the mid-tendon did not change (Kongsgaard et al. 2007; Seynnes et al. 2009). Regional hypertrophy is compatible with the mechanical demands placed on the tendon near osteotendinous junctions, where stress concentrations are the highest (Kongsgaard et al. 2007; Seynnes et al. 2009). However, findings regarding region-specific adaptations in the Achilles tendon are mixed. While some studies reported significant changes in CSA at specific regions (Bohm et al., 2014; Arampatzis et al., 2010) others observed more uniform changes across the tendon (Epro et al., 2017; Lambrianides et al., 2024).

**Table 2:** Summary of key studies that have examined region-specific tendon adaptations following exercise intervention. Table includes participant characteristics, training modalities, exercise parameters, and primary findings, with a focus on how different interventions influence hypertrophy across different regions of the tendons of lower limb muscles

Author	Participants	Training Modality	Reps × Sets	Intensity	Duration (weeks)	Frequency (days/week)	Results
Arampatzis et al. (2007)	3 YM, 8 YF	Isometric plantar flexion	4 × 5 (high strain) vs 7 × 5 (low strain)	90% MVC (high strain) 55% MVC (low strain)	14	4	[Achilles] High strain group: CSA mainly increased in MID region; Low strain group: No changes in CSA
Arampatzis et al. (2010)	11 YM	Isometric plantar flexion	12 × 5 (high strain) vs 20 × 5 (low strain)	90% MVC (high strain) 55% MVC (low strain)	14	4	[Achilles] No changes in CSA for both groups
Bohm et al. (2014)	39 YM	Isometric plantar flexion (Long Strain Duration) One-legged jumps (High Strain Rate)	Long Strain Duration: 4 × 5 vs High Strain Rate: 72 jumps × 5 sets	90% MVC (Reference & Long Strain Duration)	14	4	[Achilles] Long Strain group: CSA increased in PROX to MID region; High Strain Rate group: CSA increased towards PROX to MID and MID to DIS region
Bloomquist et al. (2013)	17 YM	Deep Squat vs. Shallow Squat	3–5 × 3–10	Progressive loads	12	3	[Patellar] No changes in CSA
Carroll et al. (2011)	16 OM, 8 OF	Bilateral knee extensor resistance training	10 × 3	~ 74% 1RM	12	3	[Patellar] Ibuprofen Group: CSA increased in DIS region; Acetaminophen Group: CSA increased in MID and DIS regions
Epro et al. (2017)	34 OF	Isometric plantar flexion	4 × 5	90% MVC	14	3	[Achilles] Uniform adaptations to CSA
Kongsgaard et al. (2007)	12 YM	Leg extension (unilateral training: one heavy-load leg, one light-load leg)	Heavy-load 10 × 8 Light-load 10 × 36	Heavy-load: 70% 1RM Light-load: Equal absolute work to heavy-leg	12	3	[Patellar] Light-Load Training: CSA increased significantly in PROX region; Heavy-Load Training: CSA increased significantly in PROX and DIS regions
Lambrianides et al. (2024)	9 YM, 3 YF	Unilateral isometric cyclic plantar flexion	3 × repetitions to failure	80% MVC	12	3	[Achilles] Uniform adaptations to CSA
Seynnes et al. (2009)	15 YM	Unilateral knee extension	4 × 10	80% 1RM	9	3	[Patellar] CSA increased in PROX and DIS regions but not in the MID region (40–50% of tendon length)

YM, Young males; YF, Young females; OF, Older females; OM, Older males; MVC, Maximal voluntary contraction; 1RM, One-repetition maximum; CSA, Cross-sectional area; PROX, Proximal region; MID, Middle region; DIS, Distal region

#### Neural adaptations

Up to now there are not many studies that have directly examined whether training adaptations in muscle activation or in motor unit discharge characteristics differ between proximal and distal regions of lower limb muscles. The available evidence comprises primarily of acute interventions (Watanabe et al. 2013b; Pincheira et al. 2021), with only one longitudinal study currently available (Narouei et al. 2022). For instance, although regional increases in proximal muscle activation have been observed following limb-loaded walking interventions in older adults (Narouei et al. 2022), these changes were not consistent across different regions or associated with improved function. Similarly, localized fatigue responses within the RF suggest region-specific neuromuscular adaptations during sustained contractions (Watanabe et al. 2013b), but these observations have not been validated under dynamic conditions. Additionally, Pincheira et al. (2021) examined the regional changes in MG activation following repeated bouts of eccentric exercise, concluding that neural adaptations were not responsible for the repeated bout effect. Collectively, these studies highlight the scarcity of the current evidence regarding region-specific adaptations in muscle activation and motor unit behavior following training interventions, underscoring a critical need for further research in this area.

### Injury and unloading

#### Musculoskeletal adaptations

Current evidence suggests region-specific adaptations in muscle architecture following injury, although findings vary considerably depending on the type of injury, and the involved structured (muscle vs tendon injury). A detailed summary of key studies examining these region-specific adaptations is provided in Table 3. For instance, following anterior cruciate ligament reconstruction greater reductions in CSA and length have been found in distal compared to proximal regions in ST (Kositsky et al. 2023; Hjaltadóttir et al. 2024). Similarly, after Achilles tendon rupture, non-uniform patterns of tendon compliance have been observed, with certain regions displaying increased compliance while others remain stiff (Khair et al. 2021). No evidence of region-specific adaptations has been found following Achilles tendinopathy, with the injured and uninjured limb presented uniform increase in CSA and anterior-posterior diameter (Nuri et al. 2017). In contrast, hamstring strain injuries present inconsistent findings, with some studies reporting no significant region-specific differences in muscle or aponeurosis adaptations (Lazarczuk et al. 2024; Yagiz et al. 2024), while others found greater increase in passive stiffness of BF in the proximal compared to the distal region (Nuñez et al. 2022). Furthermore, during unloading, region-specific reductions in CSA are commonly observed, although the affected regions differ across muscles. For instance, unloading induced greater atrophy in distal regions of the MG and ST, whereas more uniform changes were observed in other muscles such as the BF (Miokovic et al. 2012). Also, Lee et al., (2006) found that the strain distribution in the mid-aponeurosis changed significantly, whereas the distal aponeurosis strain remained relatively unchanged after 4 week of unilateral lower limb suspension. Overall, these findings emphasize the variability and limited consensus regarding region-specific adaptations post-injury or unloading, highlighting the necessity for further research to clarify their clinical relevance.

**Table 3:** Summary of key studies that have examined muscle and tendon region-specific adaptations following injury and unloading. Table includes participant characteristics, type of injury, and primary findings, with a focus on injury and unloading influence the muscle and tendon adaptations across different regions of the lower limb muscles

Author	Participants	Type of injury	Results
Injury related studies			
Hjaltadóttir et al. (2024)	7 YM, 11 YF	ACL reconstruction	[ST] Greater reduction CSA in the DIS than PROX region in INJ than UNINJ limb; [BFlh] No change in CSA
Hjortshoej et al. (2024)	57 YM	Patellar tendinopathy	[Patellar] Thickness increased across the different measurement location in the INJ than UNINJ limb. Greater increase at PROX region
Khair et al. (2021)	17 YM, 3 YF	Achilles tendon rupture	[Achilles] Injured tendons displayed non-uniform displacement patterns, with some regions showing increased compliance while others remained stiff
Khair et al. (2022)	24 YM, 4 YF	Achilles tendon rupture	[Achilles] Similar displacement pattern between different locations in INJ and UNINJ limb
Kositsky et al. (2023)	4 YM, 6 YF	ACL reconstruction	[ST] Greater reduction in muscle length and volume in distal compared to proximal compartment in INJ than UNINJ limb
Lazarczuk et al. (2024)	13 YM	Hamstring strain injury	[BFlh] Aponeurosis and muscle CSA did not present region specific adaptations in INJ than UNINJ limb
Marcon et al. (2015)	22 YM, 12YF	ACL reconstruction	[Quadriceps] CSA decreased uniformly across the different measurement regions in INJ than UNINJ limb
Morris et al. (2021)	4 YM, 11YF	ACL reconstruction	[ST] CSA decreased across all measurement regions in INJ than UNINJ limb. Greater decrease was observed at DIS region (61%), followed by MID region (46%) and then PROX region (23%); [BFlh] No change in CSA between INJ and UNINJ limb in the PROX and MID regions
Nagano et al. (2015)	6 YM	Hamstring strain injury	[ST] MT increased in PROX and MID region from rest to contraction; [BFlh] MT increased in MID region from rest to contraction, but not in PROX region
Nielsen et al. (2023)	7 YM, 3YF	Calf strain injury	[MG] FL was shorter in the DIS region in INJ than UNINJ limb, no differences were found for the MID region; PA increased more during contraction in the DIS region in INJ than UNINJ limb, no differences were found for MID region
Nuñez et al. (2022)	10 YM	Hamstring strain injury	[BFlh] Passive stiffness increased in the PROX region in INJ than UNINJ limb; No changes were observed for the DIS region
Nuri et al. (2017)	10 YM	Achilles tendinopathy (mid-portion)	[Achilles] CSA and the AP diameter increased uniformly in the tendinopathic tendon compared to the healthy tendon
Setuain et al. (2017)	30 YM, 10YF	ACL reconstruction	[BFlh ST SM] CSA decreased only at MID region for ST, no changes for DIS region and other muscles; [GRA] CSA decreased only at DIS compared to MID region; [Quadriceps] No changes in CSA
Yagiz et al. (2024)	19 YM	Hamstring strain injury	[BFlh] No differences between MID and DIS region in passive stiffness
Unloading related studies			
Franchi et al. (2022)	10 YM	Unloading	[ST] CSA decreased in DIS region, while no differences in the other measurement regions; [BFlh] CSA decreased across all measurement regions. Greater reduction in DIS region than the others; [BFsh SM] CSA decreased across all measurement regions, greater reduction in MID than DIS region
Kinugasa et al. (2010)	4 YM, 1YF	Unloading	[Achilles] Region specific increase in CSA of distal aponeurosis and tendon
Lee et al. (2006)	5 YM, 3YF	Unloading	[Soleus aponeurosis] Mid-aponeurosis showed increased strain while DIS regions remained unchanged
Miokovic et al. (2012)	9 YM	Unloading	[RF] CSA reduced at PROX region, unchanged at MID and DIS region; [Vastii] CSA reduced at PROX regions, no changes at MID and DIS regions.; [Adductor Magnus] CSA reduced at PROX region, minimal change at MID and DIS regions; [BFlh] CSA reduced at all regions, greatest at MID and DIS regions; [BFsh] CSA reduced at all regions, more at MID than DIS; [MG LG] CSA reduced at all regions, greatest at DIS region; [Tib Post/FHL] CSA reduced at MID region, minimal at PROX and DIS region; [Peroneii/TA] CSA reduced at all regions, most at PROX region

YM, Young males; YF, Young females; OF, Older females; OM, Older males; CSA, Cross-sectional area; FL, Fascicle length; PA, Pennation angle; PROX, Proximal region; MID, Middle region; DIS, Distal region; INJ, Injured; UNINJ, Uninjured; ACL, Anterior cruciate ligament; ST, Semitendinosus; BFlh, Biceps femoris long head; MG, Medial Gastrocnemius; SM, Semimenbranosus; GRA, Gracilis; BFsh, Biceps femoris short head; LG, Lateral Gastrocnemius; Tib Post, Tibialis Posterior, FHL, Flexor Hallucis Longus; TA, Tibialis Anterior; RF, Rectus Femoris

#### Neural adaptations

Pathologies related to structural or functional impairments also influence regional adaptations in muscle activation, often disrupting the normal spatial activation patterns (Falla and Gallina 2020). Changes in regional activation have also been reported following injury in the lower limb muscles, although the current evidence remains limited (Gallina et al. 2019; Mendez-Rebolledo et al. 2024). In individuals with patellofemoral pain syndrome, diminished task-specific coordination between the VL and VM has been observed, leading to a more homogeneous activation pattern that may increase joint loading asymmetries (Gallina et al. 2019). Similarly, in chronic ankle instability, peroneus longus revealed altered regional activation patterns of posterior compartments (decreased EMG amplitude), indicating an impaired neuromuscular control, which potentially limiting the functional capacity of this muscle (Mendez-Rebolledo et al. 2024). Despite these observations, the literature remains sparse and predominantly descriptive, highlighting a significant knowledge gap on how injury-induced adaptations differ regionally within lower limb muscles and emphasizing the necessity for further research in this area.

## Future directions

Practical implications drawn from existing evidence on regional differences in muscle architecture and neuromuscular adaptations are currently limited and largely hypothetical, owing to the scarcity of longitudinal and mechanistic studies in humans. Nonetheless, the current available evidence in animal models raising several important research questions that needed to be addressed. For instance, could training approaches that target distal muscle regions, often richer in fast-twitch fibers and longer sarcomeres, enhance performance in explosive tasks? Similarly, can region-specific eccentric training or targeted loading of aponeurotic and tendinous structures reduce the risk of strain injuries by improving local mechanical resilience? Finally, given the evidence of region-specific motor unit recruitment, could targeted training that promotes regional activation lead to site-specific muscular adaptations and enhance functional outcomes? Although these questions present exciting potential, they remain speculative due to limited direct human evidence. Consequently, longitudinal studies are essential to determine whether the observed region-specific differences in neuromuscular system translate into meaningful functional outcomes, and if such knowledge could inform the development of more effective training and rehabilitation programs.

## Conclusion

Existing evidence suggests that skeletal muscles exhibit regional differences at structural and neural level. These differences suggest a compartmentalized or region-specific organization that may underpin distinct functional roles. While these findings are supported by both animal and human studies, the majority of the available data remain descriptive, lacking longitudinal or mechanistic insights. Consequently, the functional relevance of these regional features remains insufficiently defined.

## Abbreviations

BF: Biceps femoris
CNS: Central nervous system
PCSA: Physiological cross-sectional area
CSA: Cross-sectional area
EMG: Electromyography
LG: Lateral gastrocnemius
MG: Medial gastrocnemius
RF: Rectus femoris
VL: Vastus lateralis
VM: Vastus medialis
ST: Semitendinosus
